# Off-loading and compression therapy strategies to treat diabetic foot ulcers complicated by lower limb oedema: a scoping review

**DOI:** 10.1186/s13047-023-00659-3

**Published:** 2023-09-06

**Authors:** Justine Tansley, Richard Collings, Jennifer Williams, Joanne Paton

**Affiliations:** 1 Torbay and South Devon NHS Foundation Trust, Torquay, UK; 2 University of Plymouth, Plymouth, UK

**Keywords:** Diabetic foot ulcer, Lower limb oedema (edema), Off-loading, Compression therapy, Wound-healing, Scoping review

## Abstract

**Background:**

Lower limb oedema is a common co-morbidity in those with diabetes and foot ulceration and is linked with increased amputation risk. There is no current guidance for the treatment of concurrent diabetic foot ulcers and lower limb oedema, leading to uncertainty around the safety and efficacy of combination approaches incorporating offloading and compression therapies.

To determine indications and contraindications for such strategies and identify any other supplementary treatment approaches, a scoping review was undertaken to map the evidence relating to off-loading and compression therapy strategies to treat both diabetic foot ulcers and lower limb oedema in combination.

**Methods:**

Following the Joanna Briggs Institute (JBI) and PRISMA – Scoping Review (ScR) guidance, this review included published and unpublished literature from inception to April 2022. Literature was sourced using electronic databases including Cochrane Library, PubMed, CINAHL, AMED; websites; professional journals and reference lists of included literature. Eligible literature discussed the management of both diabetic foot ulceration and lower limb oedema and included at least one of the treatment strategies of interest. Data extraction involved recording any suggested off-loading, compression therapy or supplementary treatment strategies and any suggested indications, contraindications and cautions for their use.

**Results:**

Five hundred twenty-two publications were found relating to the management of diabetic foot ulcers with an off-loading strategy or the management of lower limb oedema with compression therapy. 51 publications were eligible for inclusion in the review. The majority of the excluded publications did not discuss the situation where diabetic foot ulceration and lower limb oedema present concurrently.

**Conclusions:**

Most literature, focused on oedema management with compression therapy to conclude that compression therapy should be avoided in the presence of severe peripheral arterial disease. Less literature was found regarding off-loading strategies, but it was recommended that knee-high devices should be used with caution when off-loading diabetic foot ulcers in those with lower limb oedema. Treatment options to manage both conditions concurrently was identified as a research gap. Integrated working between specialist healthcare teams, was the supplementary strategy most frequently recommended. In the absence of a definitive treatment solution, clinicians are encouraged to use clinical reasoning along with support from specialist peers to establish the best, individualised treatment approach for their patients.

**Trial registration:**

Open Science Framework (osf.io/crb78).

## Introduction

The management of diabetic foot ulcers (DFU) complicated by the effects of lower limb oedema is clinically challenging. Both conditions can be complex requiring a multi-faceted treatment approach. Wound healing is often prolonged in the presence of oedema because it reduces capillary blood flow [1]. Fluid accumulation in the limbs increases wound exudate levels, raising the risk of infection and further tissue breakdown [2]. Subsequent increase in limb weight can affect mobility, cause joint and soft tissue pain and elevate the plantar pressure and tissue stress transmitted to the foot ulcer [1].

Two European prospective cohort studies [3, 4], have linked lower limb oedema with an increased risk of amputation in those with a DFU. A further retrospective cohort study found survival rates were poor, following diabetes-related leg amputations [5]. These studies are widely acknowledged and cited amongst the literature, yet are limited as they only provide an observation of the potential impact that oedema has on the outcomes of DFU. They do not introduce interventions or strategies to manage the two conditions together.

According to International guidelines, DFU often require an ‘off-loading’ intervention to relieve pressure [6]. The specific nature of an off-loading intervention varies depending on wound location and factors such as ischaemia and infection [6]. International guidance recommends a non-removable, knee-high off-loading device, such as a total contact cast, as the first-line treatment option to promote wound healing in DFU [6, 7]. Physical symptoms produced by lower limb oedema such as increased limb size or volume, wet and leaking skin, leg ulceration and eczematous skin conditions, may prohibit the use of such knee-high off-loading interventions and lead to compromise.

Alternative ankle-high off-loading devices followed by felted foam in combination with appropriately fitting footwear, are suggested as the last treatment resort [6, 7]. These may appear more suitable for a person with symptoms of lower limb oedema, but the evidence suggests that they are not as effective in treating DFU [7].

The benefit of oedema management to improve DFU outcomes is widely acknowledged [1, 2], yet it is not routinely considered as part of the standard multi-faceted approach to DFU management, where treatment of complications arising from peripheral arterial disease, neuropathy, infection and foot deformities are a priority [1, 2]. Compression therapy is considered a primary intervention in the management of lower limb oedema [8] and supported by a strong evidence base of randomised controlled trials and systematic reviews [9].

However, clinicians could be unsure how to overcome the practical challenges for the use of compression therapy when a DFU is also being managed, as this remains an area which is poorly understood [2], alongside the absence of any definitive guidance for treatment.

A scoping review method was chosen due to the broad nature of the research question and the lack of definitive randomised control trials in the area of DFU management where lower limb oedema is an added complication. This method is best suited to map the evidence base and identify any gaps in the literature [10] relating to off-loading and compression therapy strategies to manage both diabetic foot ulcers and lower limb oedema in combination.

An initial search for systematic and scoping reviews found five systematic reviews evaluating the effectiveness of various strategies to manage or enhance the healing of DFU, all of which acknowledge lower limb oedema as a risk factor [11–15] and one scoping review exploring the effect of compression bandaging on the healing of DFU [16]. None examined a multi-morbidity approach to scoping the evidence base specifically focusing on management strategies where diabetic foot ulcers and lower limb oedema co-exist.

## Review aim

The aim of the review was to map any available evidence and literature to determine the off-loading and compression therapy strategies evaluated to treat both DFU and lower limb oedema. To also further understand which strategies are not recommended for this population, identify any other supplementary treatment strategies and determine any gaps in the literature.

## Objectives of the scoping review were to establish

1. Which off-loading strategies can be used to treat DFU for people who also have lower limb oedema?
2. Which off-loading strategies are not recommended or contraindicated in the treatment of DFU for people who also have lower limb oedema?
3. Which compression therapy strategies to manage lower limb oedema can be used where a DFU is present?
4. Which compression therapy strategies are not recommended or contraindicated in the management of lower limb oedema where a DFU is present?
5. Whether any other supplementary treatment strategies can be identified from the review?
6. What are the gaps surrounding the strategies to manage DFU and lower limb oedema in combination, in the current literature?

## Methods

### Protocol and registration

A scoping review protocol was developed using the Joanna Brigg’s Institute (JBI) guidance on scoping reviews [10] and the Preferred Reporting Items for Systematic Reviews and Meta-Analysis – Scoping Review (PRISMA-ScR) checklist [17]. It is recommended that protocols are registered with research organisations to help avoid the duplication of work and encourage collaborations [10]. This protocol was prospectively registered with the Open Science Framework on 21/01/2022 available at: https://doi.org/10.17605/OSF.IO/CRB78 (Registration number: osf.io/crb78).

### Inclusion criteria

- Any information (published or unpublished) relating to DFU management with an off-loading strategy.
- Any information (published or unpublished) relating to lower limb oedema management with a compression therapy strategy.
- Any information (published or unpublished) relating to the management of a DFU and lower limb oedema, where both conditions present together.
- Literature in the context of improved outcomes: wound healing, amputation rates, infection rates, quality of life or care delivery;
- Information available in the English language (for feasibility reasons).
- Information inclusive of any geographical regions, cultural backgrounds, gender, research methods, care setting, care provider or publication date.

### Information sources

This scoping review included both published and unpublished literature. Published sources included: electronic databases such as, Cochrane, PubMed, CINAHL; Professional journals; National and International organisations and charities responsible for publishing guidance. Unpublished sources included: conference abstracts; patient and clinician advice websites; commercially available trials and information.

### Search and screening strategy

This scoping review followed the JBI’s recommended search strategy consisting of three steps [10]. (Searching took place between 10th January – 1^st^ April 2022). Two key databases (PUBMED, CINAHL) were used in a preliminary search by the first reviewer (JT) and assisted in the refining of search terms with the support of an information specialist. A second search was performed across all the information sources using the refined set of search terms, with consideration being given to alternative spellings of key words (oedema/edema/odema). A third search examined any reference lists, to identify any further literature of use. A full list of search terms can be viewed in Appendix.

The title and abstract was independently screened by two reviewers (JT, JW) on all of the literature found. A pilot screening took place to ensure both reviewers were clear and consistent with the eligibility criteria before the principle screening. Once eligible literature was determined, full text screening was carried out by the first reviewer (JT).

### Data charting and data items

A table was prepared in Microsoft Excel, adapted from a JBI template [10], to record findings from the data extraction exercise. This was used as a prompt to record any relevant findings from each piece of literature such as the treatment strategy, methods, outcomes and any other key findings. A chart for mapping the literature was developed in Microsoft Excel, linked to the objectives and eligibility criteria of the scoping review, which followed the required reporting items for scoping reviews [17]. Its purpose was to assist in identifying any relevant concepts in context with the scoping review and identify any gaps in the literature.

### Appraisal of literature

Although scoping reviews are not intended to synthesise results or require a risk of bias assessment unlike a systematic review [10], the literature was mapped against the Alper & Haynes (2016) integrated ‘6S’ levels of organisation of evidence pyramid model [18] to give an impression of the quality of the available literature and its validity to everyday clinical practice.

## Results

### Summary

**Fig. 1:**
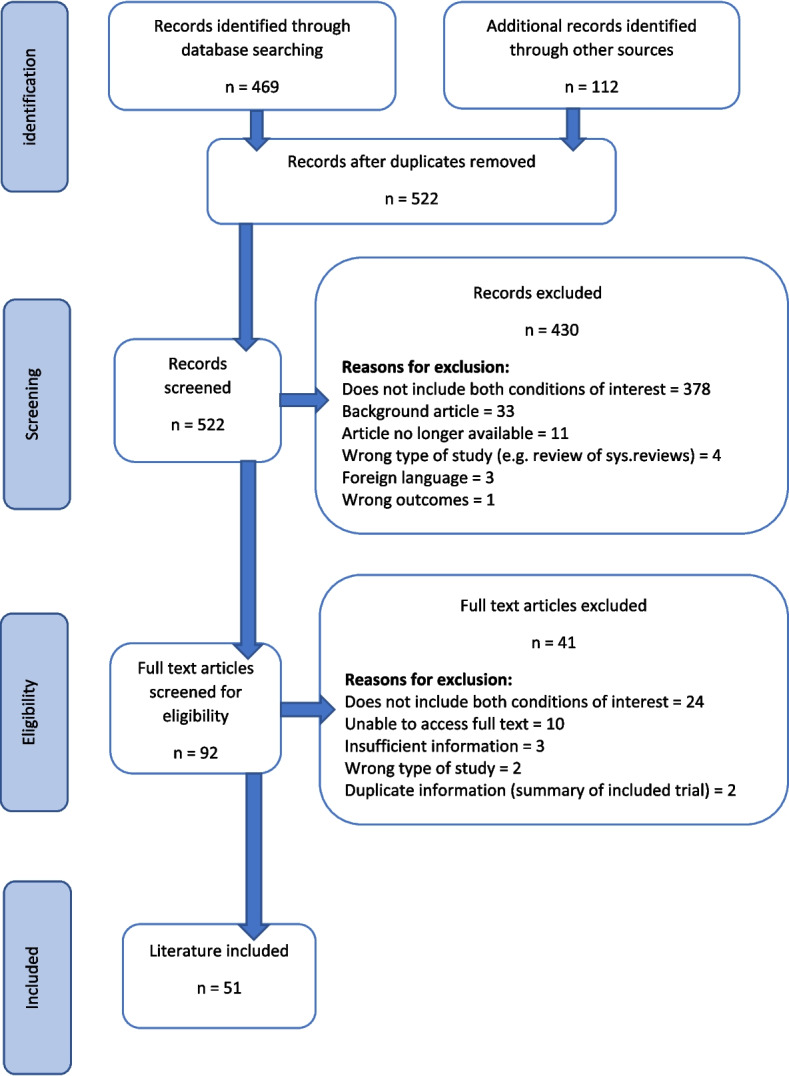
PRISMA flow diagram for the scoping review process [10, 17]

A total of 522 pieces of literature were found from all searches. Fifty-one pieces of information were included in the final scoping review as detailed in Table 1. All of the included information addressed both conditions and included at least one of the management strategies of interest. Some of the literature discussed more than one strategy. A summary of the searching and screening process is displayed in the PRISMA flow diagram in Fig. 1. Publications that did not discuss the situation where diabetic foot ulceration and lower limb oedema present concurrently, was the most common reason for exclusion at both the title and abstract screening (*n* = 378, 88%) and full text screening (*n* = 24, 59%) stages.

**Table 1:** Key management strategies identified from included literature with level of evidence

	Key Strategies identified and frequency in literature (some literature included more than one strategy)	Sources of evidence
**Compression therapy**		
Total no. of strategies = **3** *Included in 24 pieces of literature*	Compression bandaging **11 **[2, 16, 19–26] Compression hosiery/wrap devices **10 **[20, 27–35] Pneumatic compression devices **5 **[34, 36–39]	*Randomised controlled trial ****2*** [31, 36] *Prospective cohort study ****1 ***[30] *Pilot randomised controlled trial ****1 ***[35] *Scoping review ****1 ***[16] *Case study 7 *[19, 21, 22, 24, 26, 27, 37] *Foundational sources*^*a*^***12 ***[2, 20, 23, 25, 28, 29, 32–34, 38–40]
**Off-loading**		
Total no. of strategies = **7** *Included in 13 pieces of literature*	Total contact cast **5 **[2, 41–44] Knee high walking cast/boot **4 **[28, 45–47] Ankle-high walking cast/boot **2 **[27, 48] (Other devices **4**) [28, 49–51]	*Retrospective cohort study ****1 ***[41] *Case study 3 *[27, 42, 49] *Foundational sources*^*a*^***9 ***[1, 28, 43–48, 50, 51]
**Compression therapy and off-loading in combination**		
Total no of strategies = **3** *Included in 3 pieces of literature*	Ankle high boot + compression bandaging **1 **[27] Back slab cast + compression bandaging **1 **[49] General off-loading + compression bandaging **1 **[25]	*Case study2 *[27, 49] *Foundational sources*^*a*^***1 ***[25]
**Supplementary strategies**		
Total no. of strategies = **16** *Included in 20 pieces of literature*	Integrated working **5 **[20, 25, 52–54] Patient specific care plans **2 **[54, 55] Wound/limb triage tools **2 **[56, 57] (Other strategies **13**) [1, 2, 19, 20, 25, 27, 42, 45, 49, 58–62]	*National guidance documents****2 ***[52, 53] ^[[52, 53]]^ *Observational study****1 ***[59] ^[[59]]^ *Case study 5 *[19, 27, 42, 49, 62] *Foundational sources*^*a*^***12 ***[1, 2, 20, 25, 45, 54–58, 60, 61]

^a^ Foundational sources include: clinical audit and reviews, literature reviews, expert opinion pieces, conference abstracts, medical industry information

### Literature characteristics

The included literature spanned a date range of 24 years (1998 – 2022). It was produced from 13 different countries with the UK (*n* = 21, 41%) and USA (*n* = 10, 20%) being the most prevalent. 44 pieces of literature came from a published source (86%) and seven from unpublished sources (14%). Literature considered to be higher in quality such as evidence-based summaries and guidance, evidence synthesis and research studies [18] were fewer in numbers (*n* = 21, 41%). Foundational resources and unpublished literature which is considered to be lower in quality [18], was higher in numbers (*n* = 30, 59%). Details for evidence type can be viewed in Table1.

The majority of the included literature related to the use of compression therapy as a strategy to manage lower limb oedema where a DFU is present (*n* = 24, 51%). There was less information available regarding off-loading strategies (*n* = 13, 25%). Only three pieces of literature discussed the use of both an off-loading and compression therapy strategy simultaneously (6%). Nine pieces of literature solely focused on a supplementary strategy (18%), although 16 supplementary strategies were identified in total across all of the included literature. Details for these results can be viewed in Table1.

### Off-loading strategies recommended or contraindicated in the treatment of a DFU for people who also have lower limb oedema

The off-loading strategies to treat a DFU in those with lower limb oedema, mapped against the review objectives, can be viewed in Table 2. Total contact casting in the presence of lower limb oedema was most frequently discussed in the literature (*n* = 5) [1, 41–44]. This type of cast was described to primarily treat a diabetic foot ulcer by immobilising the foot and ankle and off-loading pressure from the wound area. However, appropriate use where lower limb oedema is present appeared uncertain. One retrospective cohort study [41], found that oedema was a contributory factor to adverse events in those receiving treatment for a DFU, such as the development of a new wound, infection, pain or discomfort requiring cast removal. The study found the patient population most prone to complication was those with "neuropathy and limb volume fluctuation due to both venous insufficiency and vasomotor lymphoedema”. Yet another piece of literature also suggests that the firm outer casing of the cast could be used to prevent or reduce oedema [42], although the author acknowledges that their suggestion is anecdotal. Peripheral neuropathy [43], osteomyelitis [2, 41, 44], soft tissue infection/cellulitis [2, 41, 43, 44] and varicose veins [44] were suggested contraindications across all of the literature.

**Table 2:** Information identified for off-loading strategies and mapped against review objectives

	Intervention/strategy and citation(s)	Citation(s)	Recommended or contraindicated for use in the presence of the named conditions or complications identified from the review										
			**Table key:** ✓ **Recommended** **X Not recommended** - **Unsure / insufficient information**										
			Diabetic foot ulcer	Lower limb oedema	Both diabetic foot ulcer and lower limb oedema	Severe Peripheral arterial disease (ABPI < 0.5)	Moderate peripheral arterial disease (ABPI 0.5—0.8)	Peripheral neuropathy	Soft tissue infection including cellulitis	Osteomyelitis	Leg ulcer	Lymphorrhoea	Varicose veins
Offloading devices or strategies for the management of diabetic foot ulcers	Total contact cast	Ho 2012 [2]	✓	-	-	-	-	-	**X**	**X**	-	-	-
		Riopelle 2021 [41]	✓	**X**	**X**	-	-	-	**X**	**X**	-	-	-
		Tickner 2016 [42]	✓	✓	✓	-	-	-	-	-	-	-	-
		Naude 2015 [43]	✓	**X**	**X**	**X**	-	**X**	**X**	-	-	-	-
		Whitelaw 2012 [44]	✓	-	-	**X**	-	-	**X**	**X**	-	-	**X**
	Removable knee-high walking cast/boot	Dept.Health W.Aus 2013 [45]	✓	✓	✓	**X**	-	-	-	-	-	-	-
		Oped 2022 [46]	✓	✓	✓	-	-	-	-	-	-	-	-
		Oped 2022 [47]	✓	✓	✓	-	-	-	-	-	-	-	-
		Edmonds 2008 [28]	✓	-	-	-	-	-	-	-	-	-	-
	Ankle-height removable cast	Oped 2022 [48]	✓	✓	✓	-	-	-	-	-	-	-	-
		Glynn 2021 [27]	✓	-	-	-	-	-	-	-	-	-	-
	Back slab / bi-valve cast	Dept.Health W.Aus 2013; Gurr 2006 [45, 49]	✓	✓	✓	-	-	-	-	-	-	-	-
	Scotch cast boot	Edmonds 2008 [28]	✓	-	-	-	-	-	-	-	-	-	-
	Off-loading shoe	Darco 2022 [50]	✓	✓	✓	-	-	-	-	-	-	-	-
	Heel off-loading device	Darco 2022 [51]	✓	✓	✓	-	-	-	-	-	**X**	**X**	-

Six pieces of literature discussed the use of removable walking casts or boots as detailed in Table 1. All of the literature agrees the primary purpose is to off-load pressure from the wound area [27, 45–49]. Four publications, discussed knee-high devices, of which one author advocates using the ridged nature of a knee-high device to act in reducing limb volume [45]. Yet other information advises that such a device should protect the limb from further damage by accommodating oedema rather than reducing it [46, 47]. The remaining two publications discussed the use of an ankle-high device. One case study [49] describes how a removable ankle boot was used to allow for the use of compression bandages. However, specific indications and contraindications or adverse effects were not reported.

A back-slab casting technique [49] and a Scotchcast™ boot [28], were described in two pieces of literature to treat DFU. However, there was insufficient information to determine whether these strategies could be used with oedema management strategies.

The remaining two strategies found, included an off-loading shoe [50], which is intended to be used with off-loading insoles and a heel reliever [51], used to treat a DFU occurring at the heel when a person is in the prone position. Both devices state they are designed to accommodate oedema, but not suitable for those with associated complications of oedema such as leg ulcers or lymphorrhoea.

### Compression therapy strategies recommended or contraindicated to manage lower limb oedema where a DFU is present

Compression therapy strategies to manage lower limb oedema where a diabetic foot ulcer is present, mapped against the review objectives, can be viewed in Table 3. This scoping review found eleven pieces of information across all of the literature, suggesting that compression bandaging was an effective way to reduce and manage oedema (as detailed in Table 1 and 2), which additionally could have beneficial effects on the healing of DFU [16, 19–26, 29, 40]. One scoping review [16] was found which explored the effect of compression bandaging on the healing of DFU. Compression bandaging was deemed to be safe in those without severe arterial compromise. Several case studies were found [19, 21, 22, 24, 26], all describing challenging examples where DFU management was complicated by lower limb oedema. A change was made to usual care, by introducing compression bandaging to reduce oedema and achieving a more positive outcome. Two further case studies [27, 49] also introduced an offloading intervention to treat plantar DFU in addition to compression therapy. All of the literature reported a positive change to DFU outcomes but none gave suggestions for contraindications or reports of adverse incidence.

**Table 3:** Information identified for Compression therapy strategies and mapped against review objectives

	**Intervention/strategy**	**Citation(s)**	**Recommended or contraindicated for use in the presence of the named conditions or complications identified from the review**										
			**Table key:** ✓ **Recommended** **X Not recommended** - **Unsure / insufficient information**										
			Diabetic foot ulcer	Lower limb oedema	Both diabetic foot ulcer and lower limb oedema	Severe Peripheral arterial disease (ABPI < 0.5)	Moderate peripheral arterial disease ABPI (0.5 -0.8)	Peripheral neuropathy	Soft tissue infection including cellulitis	Osteomyelitis	Leg ulcer	Lymphorrhoea	Varicose veins
**Compression therapy options or strategies for the management of lower limb oedema**	Compression bandaging (in general)	Angirasa 2006, Calianno 2007 [19, 23].	✓	✓	✓	**X**	-	-	-	-	-	-	-
	(Full)	Ho 2012, Burhan 2020, Glynn 2021, Atkin 2018, Bowering 1998, McIntosh 2009 [2, 16, 20, 22, 25, 27].	✓	✓	✓	**X**	**X**	-	-	-	-	-	-
	(Reduced)	Ho 2012, Burhan 2020, Boulton 2016, Bowering 1998, Chadwick 2006, Probst 2020 [2, 16, 21, 22, 24, 26].	✓	✓	✓	**X**	-	-	-	-	-	-	-
	Compression hosiery	Edmonds 2008, Atkin 2018, Medi 2022, Rother 2020, Simms 2010 [20, 28–30, 34, 35].	✓	✓	✓	**X**	-	-	-	-	-	-	-
		Wu 2017 [31]	✓	✓	✓	**X**	-	-	**X**	**X**	**X**	**X**	-
		Lohmann & Raucher 2022 [40].	-	✓	-	**X**	**X**	**X**	-	-	-	**X**	-
	Wrap systems	Glynn 2021, Atkin 2018, Medi 2022 [20, 27, 33].	✓	✓	✓	**X**	-	-	-	-	-	-	-
		Lohmann & Raucher 2022 [32].	-	✓	-	**X**	**X**	-	-	-	-	-	-
	Pneumatic compression	Simms 2010, Armstrong 2000, Filip 2007 [34, 36, 37].	✓	✓	✓	✓	✓	✓	✓	-	✓	✓	-
		NICE 2021 [38].	✓	✓	✓	-	-	-	-	-	-	-	-
		Oped 2022 [39].	✓	✓	✓	✓	✓	-	-	-	-	-	-

The review found 10 pieces of information across all of the literature which suggests that compression hosiery or wrap systems could be useful in managing lower limb oedema where a DFU is present [1, 20, 27, 28, 30–35] (Tables 1 and 3). A prospective study [30], and a 12-week, double blind, randomised controlled trial [31] were found, whose studies used participants with diabetes, with and without mild to moderate peripheral arterial disease, to test the safety of compression hosiery. Both studies also reported that compression hosiery was safe in the absence of severe peripheral arterial disease. However, participants with larger wounds, copious amounts of exudate and infection were excluded, suggesting their use was not considered suitable for larger, more complex wounds.

The use of pneumatic compression systems to manage lower limb oedema and improve healing of DFU was found in the literature and further suggests that it may be used even where severe peripheral arterial disease or non-revascularisable conditions are present [34, 36–39]. However, two publications cited supporting studies which acknowledge that their sample sizes were small and studies were of low methodological quality [38, 39]^.^

### Supplementary strategies identified from the review

The identified supplementary strategies to manage a DFU and lower limb oedema where both conditions present together, and mapped against the review objectives, can be viewed in Table 4. A total of 16 supplementary strategies were identified across all of the included literature (Table 4).

**Table 4:** Information identified for supplementary strategies and mapped against review objectives

	Intervention/strategy	Citation(s)	Recommended or contraindicated for use in the presence of the named conditions or complications identified from the review										
			**Table key:** ✓ **Recommended** **X Not recommended** **- Unsure / insufficient information**										
			Diabetic foot ulcer	Lower limb oedema	Both diabetic foot ulcer and lower limb oedema	Severe Peripheral arterial disease (ABPI < 0.5)	Moderate peripheral arterial disease ABPI (0.5 -0.8)	Peripheral neuropathy	Soft tissue infection including cellulitis	Osteomyelitis	Leg ulcer	Lymphorrhoea	Varicose veins
Options or strategies for the management of DFU and lower limb oedema where both conditions occur together	Integrated working	Atkin 2018, McIntosh 2009, NICE 2021, SIGN 2010, Wounds UK 2015 [20, 25, 52–54].	✓	✓	✓	✓	✓	✓	✓	✓	✓	✓	✓
	Leg elevation	Ho 2012, Atkin 2018, Hillson 2017, Park 2010 [2, 20, 58, 59].	✓	✓	✓	**X**	-	-	-	-	-	-	-
	Patient specific care plan	Wounds UK 2015 & 2018 [54, 55].	✓	✓	✓	✓	✓	✓	✓	✓	✓	✓	✓
	Dermal replacement allograft	Tickner 2016, Angrirasa 2006 [19, 42].	✓	**X**	✓	-	-	-	-	-	-	-	-
	Elbow crutches	Dept.Health W.Aus 2013, Gurr 2006 [45, 49].	✓	**X**	✓	-	-	-	-	-	-	-	-
	Exercise (non-specific)	Glynn 2021, McIntosh 2009 [25, 27].	✓	✓	✓	-	-	-	-	-	-	-	-
	(Theraband)	Gastaldi 2021 [60]	✓	✓	✓	-	-	-	-	-	-	-	-
	Manual lymphatic drainage	Kanapathy 2015, McIntosh 2009 [1, 25].	✓	✓	✓	-	-	-	-	-	-	-	-
	Weight control	Atkin 2018, Gastaldi 2021 [20, 60].	✓	✓	✓	-	-	-	-	-	-	-	✓
	Wound scoring tools	Martinez-De Jesus 2021, Raji 2016 [56, 57].	✓	✓	✓	✓	✓	✓	✓	✓	✓	✓	-
	Bed rest	Glynn 2021 [27].	**X**	**X**	**X**	-	-	-	-	-	-	-	-
	General skin care	McIntosh 2009 [25].	✓	✓	✓	-	-	-	-	-	-	-	-
	Neuromuscular taping	Kristiano 2021 [61].	✓	✓	✓	-	-	-	-	-	-	-	-
	Patient education	Hillson 2017 [58].	✓	✓	✓	✓	✓	✓	✓	✓	✓	✓	✓
	Pharmaological	Gastaldi 2021 [60].	-	✓	✓	-	-	-	-	-	-	-	✓
	Surgical	Lin 2013 [62].	✓	✓	✓	**X**	-	-	-	-	-	-	-

Integrated working, where multiple conditions such as DFU and oedema management may require input from multiple teams, was the most frequently mentioned supplementary strategy(*n* = 5) [20, 25, 52–54] and was one of the suggestions which could be applied to any clinical situation. However, this particular suggestion, despite its inclusion in two national guidance documents [52, 53], is referenced as based on expert opinion rather than scientific study. A similar suggestion is made by two best practice statements [54, 55], also based on expert opinion, which recommend that treatment plans should be specifically tailored to meet the individual needs of patient to maximise treatment quality. A clinical review piece [56] and a conference abstract [57] were found discussing the use of specifically designed wound and limb assessment and triage tools. Both tools acknowledged lower limb oedema as a risk factor to diabetic foot ulcers and suggest they could be used as a prompt to encourage oedema management as part of DFU treatment, further encouraging tailored treatment plans and integrated working.

Other suggested supplementary strategies included: Patient education [58], leg elevation [2, 20, 58, 59], elbow crutches [45, 49], exercise [25, 27, 60], weight control [20, 60], manual lymphatic drainage [1, 25], bed rest [27], skin care [25], neuromuscular taping [61], pharmacological [60] and surgical options [62]. The evidence to support these supplementary interventions came from foundational sources including case studies, literature reviews and expert opinion pieces which are considered to be of lower evidential quality [18].

## Discussion

A scoping review was carried out which aimed to establish what available off-loading and compression therapy strategies exist to manage a DFU complicated by the effects of lower limb oedema. Information from 51 pieces of literature were studied. The included studies used various outcomes to assess effectiveness and the overall level and quality of evidence was variable, making interpretation of the results difficult.

### Off-loading strategies

International guidance [6, 7] recommends that a non-removable knee-high cast, such as a total contact cast (TCC), is used as a first-line treatment to off-load a DFU, unless contraindicated. This scoping review found one retrospective cohort study which suggests that lower limb oedema may be one of these contraindications [41]. The study suggests that a TCC is not suitable for those with a DFU and lower limb oedema as an increased number of adverse events was reported in this population. It was agreed that such devices were primarily intended to assist with DFU healing, yet there were opposing arguments about their use in the presence of oedema and associated complications. Definitive direction regarding the indications and contraindications for the use of a TCC in these circumstances was lacking from the evidence.

Current guidance also recommends that a knee-high walking cast may be used as a second-line alternative if a non-removable TCC is not tolerated [6, 7]. The literature found by the review was conflicting. Some of the literature suggests that a removable knee-high walking cast should accommodate lower limb oedema for limb protection [46, 47], yet other literature supports the use of a removable pneumatic walker cast, to off-load a foot wound and reduce oedema [45]. However, both suggestions were not supported by scientific studies or other forms of evidence. There was a lack of information regarding the use of knee-high removable casts/walkers to treat a DFU where lower limb oedema was present and no discussion was found concerning appropriate use or contraindications in these circumstances.

An ankle-high removable cast is a third-line recommendation, if a knee-high cast is not tolerated or contraindicated [6, 7]. The International Working Group for the Diabetic Foot, acknowledge this recommendation in their guidance is not supported by high quality evidence [6]. The literature found by the review, suggests that an ankle-high design is intended to allow for treatment of a leg condition [27, 48], yet it is difficult to make a definite conclusion as to the suitability of this strategy to treat a DFU in the presence of lower limb oedema. No scientific studies were found demonstrating that these off-loading devices could be safely and effectively used in combination with a leg treatment such as compression therapy.

Two further strategies were found which are not included in any current guidance. They included: The use of a back-slab style cast [49], to off-load a diabetic foot ulcer and accommodate any fluctuations in lower limb oedema; a heel off-loading device [51] designed to relieve pressure from a heel wound when a person is lying prone, which may accommodate leg swelling but it is not suitable if leg wounds or exudate are present. Both strategies were not supported by scientific studies or other forms of high-level evidence.

### Compression therapy strategies

Although there is no current guidance for the use of compression therapy to manage lower limb oedema in the presence of a DFU, benefits for its use are acknowledged in the literature [16]. This scoping review found that full-strength multi-layer bandaging may be used in those without arterial compromise; reduced-strength bandaging may be used in those with reduced arterial blood supply; and a wound was unlikely to heal if there was severe arterial compromise as compression is likely to further reduce blood flow [16, 19–26, 29, 40]. Several case studies [21, 22, 24, 26, 27, 37, 42, 49] were found all sharing successful practice where DFU management was complicated by lower limb oedema. All of the case studies introduced compression bandaging to promote wound healing. However, reports of failed or ineffective cases and their circumstances were not found, leaving unanswered questions about the true safety and effectiveness of compression bandaging in these circumstances.

This review found literature which suggests that compression hosiery could be a useful way to manage lower limb oedema where a DFU is present [1, 20, 27, 28, 30–35]. A prospective study [30] and a 12-week, double blind, randomised controlled trial [31], used participants with diabetes, with or without mild to moderate peripheral arterial disease, to test the safety of compression hosiery. Both studies reported that there was no effect on arterial blood supply when hosiery was worn and after removal. Participants with DFU were included in the studies, but the effect on which, was not included as an outcome measure. It is therefore unknown the effect compression hosiery has on the outcomes of DFUs. Participants with large wounds, copious amounts of exudate and infection were excluded, which suggests this strategy may not be appropriate for those with more severe complex wounds.

This review found literature which suggests the use of pneumatic compression to manage lower limb oedema where a diabetic foot ulcer was also present [34, 36–39]. Wound healing and prevention of major amputation were the main outcomes of interest. The majority of the literature agreed that pneumatic compression could be used to promote healing in wounds of any aetiology, including in those with severe peripheral arterial disease where re-vascularisation is not possible. However, the literature acknowledges the supporting evidence to be of low methodological quality.

### Supplementary strategies

This scoping review found 16 supplementary strategies to manage a DFU and lower limb oedema where both conditions present together. Integrated working [20, 25, 52–54], patient specific treatment plans [54, 55] and the use of wound and leg assessment tools [56, 57] was popular in expert opinion. The rationale for these three strategies was they could be applied to any clinical situation including where complex co-morbidities exist which impact the lower limb, used to improve the quality of treatment planning and subsequent care and outcomes. However, all of the supplementary strategies found by this scoping review, lacked a scientific basis to support their use in a combination management approach of a DFU and lower limb oedema.

### Implications for practice and future research

This scoping review offers some insight into the available strategies to treat both a DFU and lower limb oedema when they present together and the evidence to support their safe and effective use. It would appear that more scientific evidence is required to determine which off-loading strategy would be the most suitable for use where lower limb oedema is present or if a concurrent oedema management strategy were being considered. Clear guidance on the indications and contraindications for the use of such off-loading strategies in these circumstances would also be welcomed. To further understand whether compression bandaging or hosiery is a suitable strategy to manage DFU complicated by the effects of lower limb oedema, more scientific evidence is required investigating the effect compression therapy has on DFU outcomes such as wound healing, infection rates and amputation rates. Further scientific evidence is needed to support the suggestions that integrated working, tailored treatment plans and wound assessment tools can be used as a strategy to improve the outcomes of DFU complicated by the effects of lower limb oedema.

Despite the review being unable to give definitive off-loading and compression therapy treatment solutions, clinicians should still strive to provide the best treatment strategy to manage a DFU where lower limb oedema is also a complicating feature. Whilst considering the information found from this review, clinicians should use their clinical reasoning skills to contemplate: the physiological differences and complications presenting in each individual patient; the purpose and intended outcome of treatment; whilst encouraging collaborative working with specialist teams, to find the most suitable treatment approach.

### Review limitations

The majority of the literature found by this review was published in the UK, followed by other western world countries such as the USA and Australia. This could mean that this scoping review is only applicable and relatable to healthcare in these countries. Furthermore, the literature did not consider different racial, ethnic and cultural behaviours and beliefs. This scoping review only included literature which was available in the English language for feasibility reasons. It is known that three pieces of literature had to be excluded at the screening stage as only the abstract was translated into English but not the full text. It is possible that other available literature may have been excluded at the search stage if the abstract was not in English.

The review found that the literature relating to oedema management with compression therapy was not explicit in describing the location or predominating aetiology of concurrently presenting DFU. Likewise, although the off-loading devices discussed in the review were clear their purpose was to relieve pressure from a plantar wound, further information about off-loading wounds at other locations of the foot, where lower limb oedema was a complication, was not found. This identified gap in the literature makes it difficult for the review to make suggestions on the management strategies relating to specific DFU complexities or locations on the foot, when lower limb oedema is an added complication.

## Conclusions

This scoping review discovered that lower limb oedema and diabetic foot ulceration was recognised as a common challenge. However, there is insufficient evidence to suggest definitively which off-loading strategies may be used to treat a diabetic foot ulcer complicated by the effects of lower limb oedema.

Limited evidence was found to suggest that a total contact cast may be contraindicated in those with a diabetic foot ulcer and lower limb oedema. In addition, the findings from the literature identified that an ankle-high off-loading device in combination with a compression therapy intervention, is an approach with potential that warrants further research and investigation.

This scoping review has found evidence to support the use of compression bandaging to treat lower limb oedema in the presence of a diabetic foot ulcers, but only where severe peripheral arterial disease can first be excluded. Compression garments such as hosiery, may be useful to manage oedema but only when a foot ulcer is not too large or complicated.

Of the sixteen supplementary strategies identified, none were supported by high quality evidence. Expert clinical opinion, most frequently suggested better integrated working between teams, would result in better foot health outcomes for the person with diabetes when both conditions occur together.

## Data Availability

The information used and analysed during the current study are available from the corresponding author on reasonable request.

## Appendix

**Table 5:** Table of search terms, including Boolean operators

Population	Concept	Context
Diabetes; Diabetes mellitus; Diabetic foot; Diabetic foot ulcer/ulceration; Diabetic foot disease; DFU **OR** Lower limb / leg + oedema/edema/odema, pitting/non-pitting oedema, oedematous Leg/limb swelling; Ankle swelling; Lymphoedema/ Lymphedema	Off-loading/pressure relief/pressure relieving + boot, shoe, walker, insole, device, intervention/method/strategy, contraindication Total contact cast; TCC; Semi-compressed felt **OR** Compression + Therapy, Bandaging, Hosiery/elastic stocking, Wrap, Garment, Sock, Device, Intervention/method/strategy, Contraindications	Outcomes: Health related: Heali* Wound healing; Amputation/rates; Infecti* Infection rates; Death/mortality/mortality rate Personal: Quality of life; Patient experience; Patient satisfaction Organisational: Care/Service delivery; Cost effectiveness; Hospital admissions; Length of hospital stay

*denotes multiple character searching was used to broaden results

## Abbreviations

DFU: Diabetic foot ulcer(s)
JBI: Joanna Brigg’s Institute
PRISMA-ScR: Preferred reporting items for systematic reviews and meta-analysis Scoping reviews
TCC: Total contact cast
